# Modulation of CRTh2 expression on allergen‐specific T cells following peptide immunotherapy

**DOI:** 10.1111/all.13867

**Published:** 2019-06-07

**Authors:** Christopher D. Rudulier, Elena Tonti, Eddie James, William W. Kwok, Mark Larché

**Affiliations:** ^1^ Department of Medicine, Division of Clinical Immunology & Allergy McMaster University Hamilton Ontario Canada; ^2^ Benaroya Research Institute at Virginia Mason Seattle Washington; ^3^ Department of Medicine, Division of Respirology Firestone Institute for Respiratory Health, The Research Institute at St. Joe’s Hamilton Ontario Canada; ^4^ Present address: Department of Medicine, Division of Respirology, Critical Care and Sleep Medicine University of Saskatchewan Saskatoon Saskatchewan Canada

**Keywords:** chemokines, immunotherapy and tolerance induction, immunotherapy vaccines and mechanisms, T cells, vaccines

## Abstract

**Background:**

Allergen immunotherapy using synthetic peptide T‐cell epitopes (Cat‐PAD) from the major cat allergen Fel d 1 has been shown, in allergen exposure studies, to significantly reduce symptoms of allergic rhinoconjunctivitis in cat‐allergic subjects. However, the immunological mechanisms underlying clinical benefit remain only partially understood. Since previous studies of whole allergen immunotherapy demonstrated a reduction in the frequency of allergen‐specific (MHC II tetramer^+^) CD4^+^ T cells expressing the chemokine receptor CRTh2, we assessed the impact of Cat‐PAD on the frequency and functional phenotype of Fel d 1‐specific CD4^+^ T cells.

**Methods:**

Using before and after treatment samples from subjects enrolled in a randomized, double‐blind, placebo‐controlled trial of Cat‐PAD, we employed Fel d 1 MHC II tetramers and flow cytometry to analyze the expression of chemokine receptors CCR3, CCR4, CCR5, CXCR3, and CRTh2, together with markers of memory phenotype (CD27 and CCR7) on Fel d 1‐specific CD4^+^ T cells.

**Results:**

No statistically significant change in the frequency of Fel d 1‐specific CD4^+^ T cells, nor in their expression of chemokine receptors or memory phenotype, was observed. However, a significant reduction in cell surface expression of CRTh2 was observed between the placebo and active groups (*P* = 0.047).

**Conclusions:**

Peptide immunotherapy with Cat‐PAD does not significantly alter the frequency or phenotype of Fel d 1‐CD4^+^ T cells, but may decrease their expression of CRTh2.

## Graphical abstract

Peptide immunotherapy with Cat‐PAD reduces the surface expression of Chemoattractant receptor‐homologous molecule expressed on Th2 cells (CRTh2) on Fel d 1‐specific T cells. Neither the frequency, nor memory phenotype of Fel d 1‐specific T cells in the blood were affected by peptide immunotherapy for cat allergy. Reduced expression of CRTh2 may modulate recruitment to sites of allergen exposure.

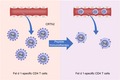

## 1 INTRODUCTION

Allergic rhinoconjunctivitis and asthma remain a significant health concern in industrialized nations despite the widespread availability of pharmacotherapy. While pharmacotherapy can effectively alleviate disease symptoms, it does not address the underlying pathogenesis of the disease, thereby necessitating continuous therapy. Thus, a safe and effective disease‐modifying treatment that addresses the underlying immunological causes of allergic inflammation is required.

Allergen‐specific immunotherapy (SIT) is the only therapy that corrects the dysregulated immune response that characterizes allergic inflammation. Traditionally, SIT involves administering the intact allergen for a period lasting from a few months to a few years. SIT has been shown to ameliorate allergic symptoms with relief lasting longer than the treatment period.^1^, ^2^ While the ability of SIT to ameliorate allergic symptoms is well documented, so too are the adverse events that can arise from administering intact proteinaceous allergens to allergic subjects.^3^, ^4^ The adverse events can be life‐threatening and stem from the ability of allergens to cross‐link allergen‐specific IgE molecules on the surface of effector cells (such as basophils and mast cells). An alternative to SIT is the use of peptides containing CD4^+^ T‐cell epitopes derived from clinically important allergens.^3^ Peptide immunotherapy performed with short peptides is associated with fewer IgE‐mediated adverse events than observed in conventional SIT, since short peptides have a markedly reduced ability to cross‐link IgE on basophils (and by inference mast cells).^5^ Peptide immunotherapy has been shown to effectively ameliorate symptoms of allergic disease in both mice and humans.^2^, ^5^, ^6^, ^7^, ^8^, ^9^

Targeting allergen‐specific Th2 CD4^+^ T cells is a rational immunotherapeutic strategy as allergen‐specific Th2 cells play an important role in the development, maintenance, and exacerbation of allergic airway disease. Mouse models have demonstrated that Th2 cells are important for the class switching of allergen‐specific B cells to IgE^10^ and that Th2 cells may also facilitate the maintenance of plasma cells.^11^ In addition, the Th2 cytokines IL‐4, IL‐5, and IL‐13 have been implicated in goblet cell hyperplasia and mucus production, induction of airway hyperresponsiveness (AHR) and the recruitment of eosinophils to the airways.^10^ Mouse models involving the adoptive transfer of Th2 cells,^12^, ^13^, ^14^ or the acute depletion of CD4^+^ T cells prior to challenge,^15^, ^16^ have demonstrated that CD4^+^ T cells play a significant role in inducing allergic airway disease.

Sensitization to cats is one of the most common types of allergic sensitization and is strongly associated with the development of asthma.^17^, ^18^ In addition, children allergic to cats, as compared to other allergens, are more likely to develop severe asthma.^19^ The principal cat allergen is Fel d 1 and peptides from Fel d 1 containing T‐cell epitopes (Cat‐PAD) capable of binding to commonly expressed class II HLA molecules have been identified and used to treat cat‐allergic subjects.^5^

The mechanisms responsible for improvements in clinical symptoms in cat‐allergic subjects remain incompletely understood. Previous studies of subcutaneous immunotherapy (SCIT), employing whole allergen extracts have described a shift from a Th2 signature to a Th1 signature following treatment,^20^ while others have described the generation of putative regulatory T‐cell populations.^21^, ^22^ Recently, deletion of allergen‐specific T cells, and in particular terminally differentiated CD27^−^ Th2 cells, has been described.^7^, ^23^, ^24^

Conceptually, altering the chemokine receptor profile of allergen‐specific T cells could prevent the recruitment of these cells to sites of allergic inflammation. Chemokine receptors play a critical role in the trafficking of T cells and other leukocytes.^21^ The chemokine expression profile of CD4^+^ T cells has been shown to correlate with their function. For example, CCR3, CCR4, and CRTh2 have been shown to be preferentially expressed by Th2 T cells, while CXCR3 and CCR5 are preferentially expressed by Th1 T cells. In regard to the T cell memory populations, there are several different types, including central memory T cells (T_CM_), effector memory T cells (T_EM_), and an intermediate population termed transitional memory T cells (T_TM_). T_CM_ cells express CCR7 while T_TM_ and T_EM_ cells do not.^22^ It has also been reported that memory T cells can be divided by their expression of CD27. CD45RA^−^CD27^+^ cells behave as T_CM_ (or T_TM_) cells in that they do not readily proliferate or secrete cytokine upon stimulation.^25^ In contrast, CD45RA^−^CD27^−^ cells do proliferate and secrete cytokines in response to antigen, which is characteristic of T_EM_ cells.^25^ The role of memory T cells in allergy has been the subject of a number of excellent reviews.^26^, ^27^

In the present mechanistic study, we sought to advance our understanding of the mechanism(s) governing the clinical impact of immunotherapy with Cat‐PAD by measuring the frequency of Fel d 1‐specific CD4^+^ T cells, their memory status, and their expression of Th1, and Th2‐associated chemokine receptors. The hypothesis for this study was that peptide treatment would alter the chemokine receptor profile of Fel d 1‐specific CD4 T cells. We isolated peripheral CD4^+^ T cells from cat‐allergic, asthmatic subjects, before and after treatment with Cat‐PAD and focused our analysis on CD4^+^ T cells using Fel d 1 MHC class II tetramers. Our analysis provides new insight into the mechanisms of action of peptide immunotherapy and highlights key differences with respect to whole allergen immunotherapy approaches.

## 2 MATERIALS AND METHODS

### 2.1 Ethics

The study was conducted in accordance with the Declaration of Helsinki and the International Conference on Harmonization (ICH) guidelines on Good Clinical Practice (GCP). The mechanistic study reported here received approval from the McMaster University/Hamilton Health Sciences Research Ethics Board.

### 2.2 Subjects

The clinical trial providing samples for this study were registered at ClinicalTrials.gov (identifier: NCT00867906). The clinical trial received approval from Institutional Review Board Services (Aurora, Ontario, Canada). All subjects provided written, informed consent to participate in the clinical trial and to provide blood samples for mechanistic studies. The subjects were male or female, aged 18‐65, with a history of controlled allergic asthma as defined in GINA (2007) on exposure to cats for at least 1 year. The subjects had a reliable history of rhinoconjunctivitis on exposure to cats for at least 1 year. In addition, the recruited subjects displayed a positive skin prick test to cat allergen with a wheal diameter at least 3 mm larger than the negative control, and a late‐phase skin reaction of >25 mm in diameter to cat allergen, eight hours after intradermal injection. The subjects were randomly assigned to the treatment and placebo groups. The primary objective of the clinical study from which the samples were obtained was to evaluate the safety and tolerability of multiple intradermal injections of Cat‐PAD in cat‐allergic subjects with controlled asthma. Secondary clinical surrogate outcomes included changes in the magnitude of the early‐phase and late‐phase skin response to intradermal allergen challenge, and change in tolerated allergen dose in a conjunctival provocation.

In total, 80 subjects were screened between March 2009 and October 2009; of these, 28 failed screening. The remaining 52 subjects were randomized with 45 subjects completing the clinical component of the study. Seven subjects withdrew from the study (4 withdrew consent; 2 withdrew for use of a prohibited medication, and 1 was lost to follow‐up). For the mechanistic studies described herein, only 13 placebo‐treated subjects and 12 Cat‐PAD‐treated subjects were analyzed as MHC class II tetramers were not available for the other trial participant samples.

### 2.3 Peptide treatment

Subjects received either Fel d 1 synthetic peptides (sequences disclosed in ref.^5^) or placebo. Fel d 1 synthetic peptides were administered intradermally at doses of 3 nmol, each subject received 8 doses, with each dose separated by 2 weeks (±2 days). The clinical study duration for each individual was up to 35 weeks. The placebo product comprised the vehicle used to formulate the Fel d 1 synthetic peptides. Its appearance was identical to the active study medication, and it was presented frozen in individual vials. Fel d 1 synthetic peptides are an equimolar mixture of seven peptides, whose individual sequences are derived from Fel d 1. The study medication was supplied as a frozen concentrate. The frozen concentrate containing the peptides at 200 nmol/mL was provided as 0.6 mL fills and was diluted with placebo to prepare the 3 nmol dose. The peptides were synthesized by Bachem (Switzerland) according to current Good Manufacturing Practice (GMP) and formulated, filled, and finished by Nova Laboratories (UK) also according to current GMP.

### 2.4 Tetramers

Recombinant HLA‐DR proteins were generated as previously described.^28^ Briefly, each HLA‐DR was purified from the supernatants of transfected insect cells, biotinylated, and dialyzed into 0.1 mol/L phosphate buffer. Biotinylated monomer was loaded with 0.2 mg/mL of peptide by incubating at 37°C for 72 hours in the presence of 2.5 mg/mL n‐octyl‐b‐D‐glucopyranoside and 1 mmol/L Pefabloc SC protease inhibitor (Sigma‐Aldrich) and then conjugated using R‐PE streptavidin (Biosource International) at a molar ratio of 8 to 1. Fel d 1 tetramers were selected based on our prior studies.^29^ Specifically, a panel of 14 tetramers were used in this study; HLA‐DRB1*01:01 (2 tetramers), HLA‐DRB1*03:01 (3 tetramers), HLA‐DRB1*04:01 (1 tetramer), HLA‐DRB1*07:01 (1 tetramer), HLA‐DRB1*09:01 (3 tetramers), HLA‐DRB1*11:01 (1 tetramer), HLA‐DRB1*13:01 (1 tetramer), HLA‐DRB5*01:01 (2 tetramers). Each subject was typed for their expression of HLA‐DRB alleles using a high‐resolution HLA DNA typing kit (One Lambda). The appropriate tetramer(s) were selected based on the subjects’ expression of these HLA‐DRB alleles.

### 2.5 Isolation of Fel d 1‐specific CD4^+^ T cells

Blood was taken from subjects at baseline (week 0) and 10‐14 weeks after the final injection (week 24‐28). PBMCs were isolated using a density gradient centrifugation and cryopreserved with RPMI media supplemented with 10% DMSO, 20% fetal bovine serum. Cryopreserved PBMCs were thawed (>90% viability) and CD4^+^ T cells were purified using magnetic selection with a negative selection cocktail according to the manufacturer's instructions (StemCell Technologies). Purified CD4^+^ T cells were incubated with 50 nmol/L dasatinib (to prevent T‐cell receptor downregulation; Cedarlane) at 150 × 10^6^/mL in RPMI media supplemented with 10% fetal calf serum, 100 U/mL penicillin (Invitrogen), and 100 μg/mL streptomycin (Invitrogen) for 20 minutes at 37°C and 5% CO_2_. Fifteen µg of the appropriate phycoerythrin (PE)‐labeled tetramer was added and incubated for another 2 hours with gentle mixing after 1 hour. The tetramer positive cells were then isolated using anti‐PE magnetic beads according to manufacturer's instructions (StemCell Technologies). Calculation of the frequencies of tetramer^+^CD4^+^ T cells used the equation:

$$\frac{\left(\%\;\mathrm{tetramer}\;\mathrm{positive}\;\mathrm{cells}\;\times \;\#\;CD4\;\mathrm{cells}\;\mathrm{in}\;\mathrm{PE}\;\mathrm{positive}\;\mathrm{fraction}\;\right)\;}{\;100}\;\;\;÷\;\mathrm{total}\;\#\;\mathrm{of}\;CD4\;\mathrm{cells}$$

### 2.6 Flow cytometry and analysis

Purified tetramer positive cells were incubated simultaneously with fluorescently labeled antibodies specific for CD4, CD8, CD14, CD19, CXCR3, CCR3, CCR4, CCR5, CCR7, CD27, CD45RA, CRTh2 (BD biosciences) in a 100 µL Cell Staining Buffer (BioLegend) for 30 minutes at 4°C. FMO controls were used for gating. Cells were then washed and run on a LSRII flow cytometer (BD biosciences). Gates were established using fluorescence minus one (FMO) controls. The data were analyzed using FlowJo (Tree Star). CD8+, CD14+, and CD19+ cells were excluded from the analysis (dump gate). The use of a dump gate increases the confidence in relatively rare events, such as tetramer+ CD4^+^ T cells.^30^, ^31^

### 2.7 Statistical analysis

Statistical analysis was performed using SPSS and GraphPad Prism. A paired t test was used to compare pre‐ and posttreatment in the placebo and active groups. A repeated measures ANOVA was used to compare within‐subject factors (ie, pretreatment vs posttreatment) and between‐subject factors). All data sets were complete and did not contain missing values.

## 3 RESULTS

### 3.1 Treatment with Cat‐PAD does not affect the frequency of Fel d 1‐specific CD4^+^ T cells

**Figure 1:**
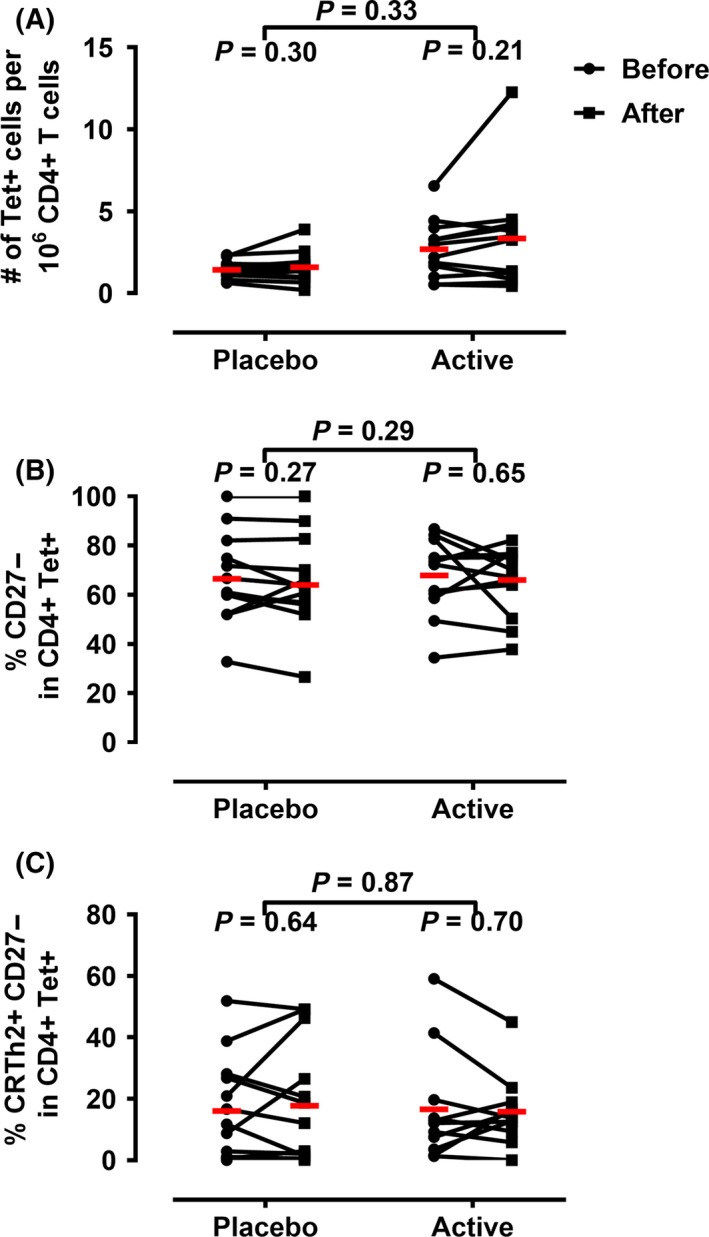
Frequency of allergen‐specific CD4^+^ T cells and terminally differentiated Th2 subsets before and after treatment with Cat‐PAD or placebo. Fel d 1‐specific CD4^+^ T cells were identified and enumerated by flow cytometry using HLA‐DR‐matched MHC class II tetramers and phenotypic markers; CD27 as a marker of differentiation status and CRTh2 as a marker of effector Th2 cells. A, Frequency of Fel d 1‐tetramer^+^ T cells per 10^6^ CD4^+^ T cells. B, Percentage of CD27^−^ cells among CD4^+^ tetramer^+^ T cells. C, Percentage of CRTh2^+^CD27^−^ cells among CD4^+^ tetramer^+^ T cells. Individual paired data from n = 12 (placebo) and n = 13 (active). Differences over time were analyzed using the paired *t* test (A, B) and the Wilcoxon signed rank test (C). The difference between the placebo and active group was assessed by RM‐ANOVA

First, we assessed the frequency of all Fel d 1‐specific CD4^+^ T‐cells pre‐ and posttherapy, regardless of phenotype. Subsequently, we specifically examined changes in subsets of allergen‐specific CD4+ T cells (CD27^−^ and CRTh2^+^) that have been shown to be absent following whole allergen immunotherapy. In contrast to previous studies of SIT, we did not observe a significant change in the frequency of tetramer^+^ CD4^+^ T cells (Figure 1A), percentage of tetramer^+^CD27^−^ (Figure 1B), or percentage of tetramer^+^CRTh2^+^ CD4^+^ T cells (Figure 1C) following treatment, suggesting that the mechanisms of action of Cat‐PAD do not involve the deletion of allergen‐specific CD4^+^ T cells.

### 3.2 Peptide immunotherapy does not alter Fel d 1‐specific CD4^+^ memory T‐cells subsets

**Figure 2:**
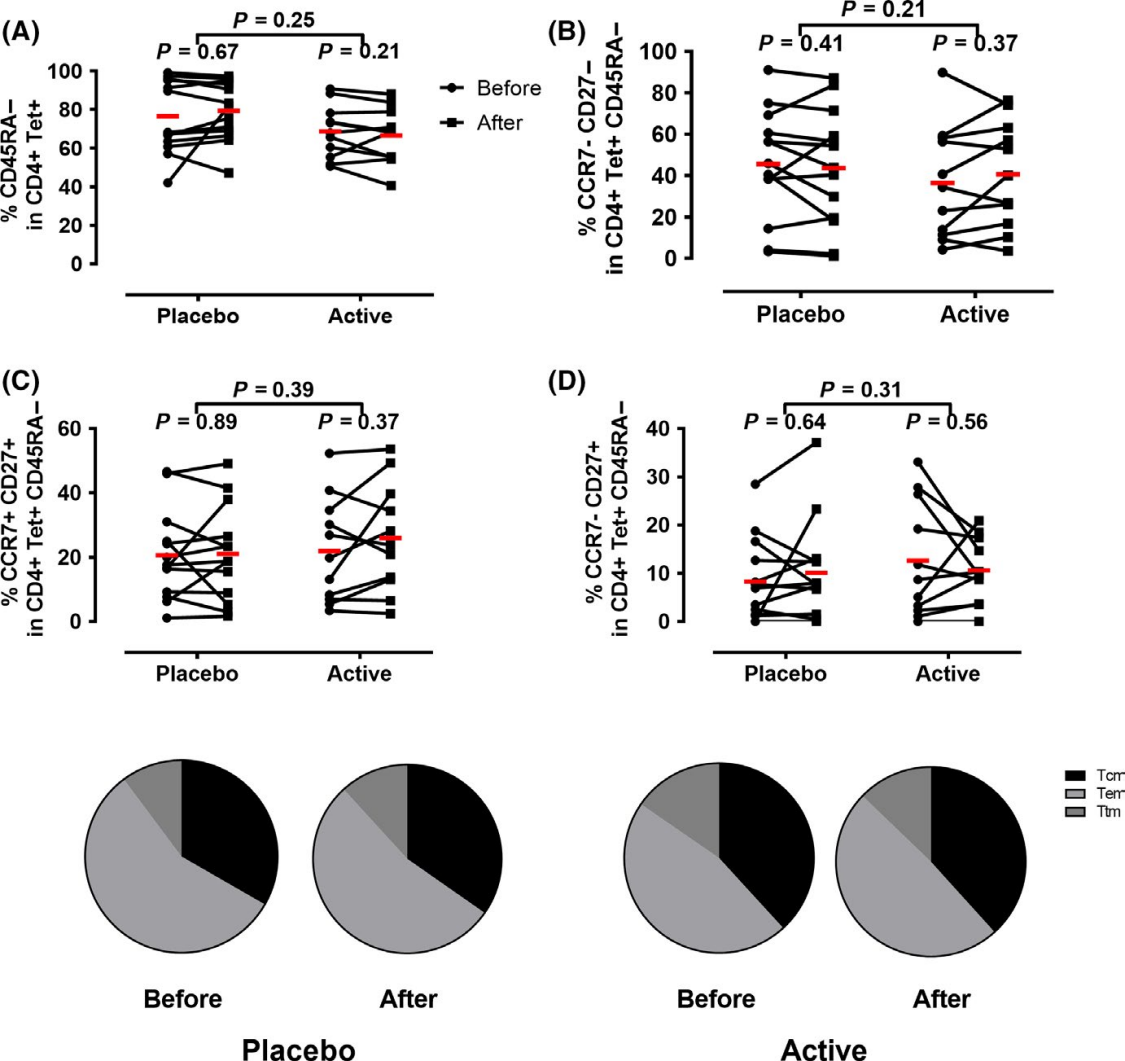
Frequency of allergen‐specific memory CD4^+^ T cell subsets before and after treatment with Cat‐PAD or placebo. A, Percentage of memory (CD45RA^−^) T cells among CD4^+^ tetramer^+^ T cells. B, Percentage of effector memory phenotype among memory CD4^+^ tetramer^+^ T cells. C, Percentage of central memory phenotype among memory CD4^+ ^tetramer^+^ T cells. D, Percentage of transitional memory phenotype among memory CD4^+^ tetramer^+^ T cells. Differences over time were analyzed using the paired *t* test. E, Relative proportions of central memory (T_CM_), effector memory (T_EM_) and transitional memory (T_TM_) within the CD4^+^ tetramer^+^ population before and after treatment. Individual paired data from n = 12 (placebo) and n = 13 (active). The difference between the placebo and active group was assessed by RM‐ANOVA

To determine if peptide immunotherapy affected memory CD4^+^ T‐cell subsets, we quantified the percentage of CD45RA^−^CD4^+^tetramer^+^ cells before and after treatment. As shown in Figure 2 (and Figure S1), peptide immunotherapy did not alter the percentage of total allergen‐specific memory T cells (CD4^+^tetramer^+^CD45RA^−^), T_EM_ (CD4^+^tetramer^+^CD45RA^−^ CCR7^−^CD27^−^), T_CM_ (CD4^+^tetramer^+^CD45RA^−^ CCR7^+^CD27^+^), or T_TM_ (CD4^+^tetramer^+^CD45RA^−^ CCR7^−^CD27^+^).

### 3.3 Peptide immunotherapy does not alter the percentage of allergen‐specific T cells expressing individual chemokines receptors

**Figure 3:**
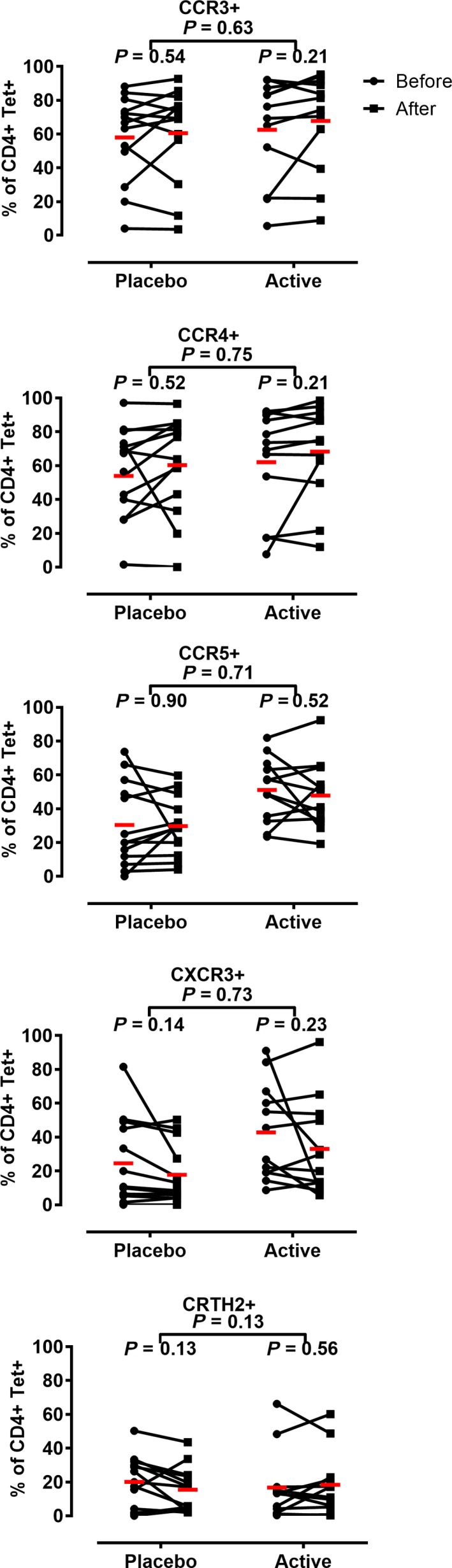
Chemokine receptor expression by allergen‐specific CD4^+^ T cells. Panels show the % of CD4^+^ tetramer^+^ T cells staining positive for chemokine receptors CCR3, CCR4, CCR5, CXCR3, and CRTh2, before and after treatment with Cat‐PAD or placebo. Individual paired data from n = 12 (placebo) and n = 13 (active). Differences over time were analyzed using the paired t test. The difference between the placebo and active group was assessed by RM‐ANOVA

Next, we investigated the effect of treatment upon the percentage of tetramer^+^ CD4^+^ T cells expressing individual Th1‐ and Th2‐associated chemokine receptors: Th1 (CCR5, CXCR3), Th2 (CCR3, CCR4, CRTh2). We found no change in the percentage of tetramer^+^ cells expressing any individual chemokine receptor (Figure 3). Similarly, no changes were observed following analysis of multiple chemokine receptors (data not shown). We conclude that peptide immunotherapy with Cat‐PAD is not associated with a change in the frequency of allergen‐specific T cells expressing any of the chemokine receptors analyzed in this study (Figure 3).

### 3.4 Treatment with Fel d 1 synthetic peptides does not alter the proportions of allergen‐specific T Th1 and Th2 chemokine receptor phenotypes

**Figure 4:**
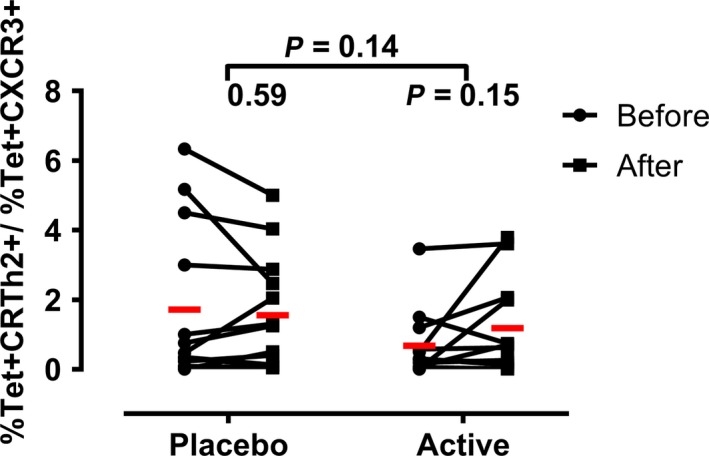
The effect of peptide immunotherapy on the ratio of CRTh2^+^tetramer^+^ T cells to CXCR3^+^tetramer^+^ T cells. Modulation, by peptide immunotherapy, of the ratio of allergen‐specific (tetramer^+^) Th2:Th1 T cells was modeled employing CRTh2 and CXCR3 as representative Th2 and Th1 markers, respectively. Individual paired data from n = 12 (placebo) and n = 13 (active). Differences over time were analyzed using the paired t test. The difference between the placebo and active group was assessed by RM‐ANOVA

Specific immunotherapy has been shown to shift the phenotype of the allergic response from Th2 to Th1.^20^, ^24^, ^32^ It is generally accepted that Th1 and Th2 cells differ in their expression of chemokine receptors. Th2 cells predominantly express CCR3, CCR4, and CRTh2, while Th1 cells predominantly express CXCR3 and CCR5. We employed representatives of these surrogate markers to assess whether treatment with Cat‐PAD affected the Th1/Th2 nature of the T‐cell response Fel d 1 by comparing the ratio of tetramer^+^ T cells expressing CXCR3 to those expressing CRTh2. As shown in Figure 4, peptide immunotherapy did not affect the ratio of tetramer^+^CXCR3^+^ to tetramer^+^CRTh2^+^ and therefore likely does not affect the overall Th1: Th2 phenotype of the response.

### 3.5 Peptide immunotherapy modulates levels of surface expression of chemokine receptors on allergen‐specific T cells

**Figure 5:**
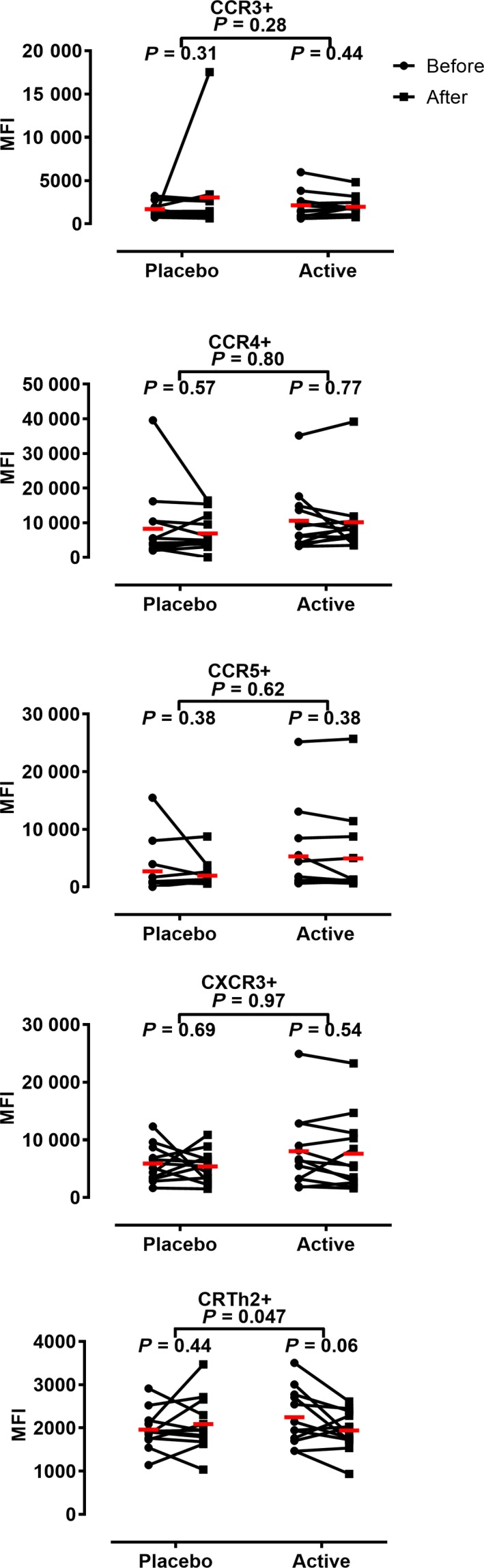
Density of surface expression of chemokine receptor by allergen‐specific T cells. Panels show the median fluorescence intensity (MFI) of tetramer^+^ CD4^+^ T cells staining positive for chemokine receptors CCR3, CCR4, CCR5, CXCR3, and CRTh2, before and after treatment with Cat‐PAD or placebo. Individual paired data from n = 12 (placebo) and n = 13 (active). Differences over time were analyzed using the paired t test. The difference between the placebo and active group was assessed by RM‐ANOVA

In addition to measuring the frequency of allergen‐specific CD4^+^ T cells expressing individual chemokine receptors, we also quantified the intensity of chemokine receptor expression by measuring median fluorescence intensity (MFI) of staining. As demonstrated in Figure 5 (and Figure S2), no changes in receptor intensity were observed for any chemokine receptor on allergen‐specific T cells following treatment with placebo. In contrast, subjects receiving treatment with Cat‐PAD showed a trend toward downregulation of CRTh2 (*P* = 0.06) which was significant when comparing the change in MFI between subjects in the placebo vs active group (*P* = 0.047) (Figure 5).

## 4 DISCUSSION

The objective of this mechanistic study was to investigate potential mechanisms of action of peptide immunotherapy when used to treat cat‐allergic subjects. The peptide mixture employed in this study (Cat‐PAD) has been shown to significantly reduce symptoms of allergic rhinoconjunctivitis in an allergen exposure chamber setting.^2^ However, the surrogate clinical outcome measures (skin early‐ and late‐phase responses to allergen challenge, and conjunctival challenge) employed in the clinical component of the current study did not demonstrate a treatment effect. However, the clinical study was designed to assess safety and tolerability and the clinical surrogate markers were secondary outcomes. Cat‐PAD was also evaluated in a phase 3 field study in cat‐allergic subjects who lived with a cat. The trial failed to demonstrate a treatment effect and was associated with an unusually high placebo response rate (approximately 60%), which may indicate a suboptimal study design.

We focused on allergen‐specific CD4^+^ T cells in the peripheral blood, and asked whether the frequency of these cells, their patterns of chemokine receptor expression, or memory T cell markers were changed. We isolated peripheral CD4^+^ T cells from cat‐allergic subjects, before and after treatment with Cat‐PAD. We were able to analyze Fel d 1‐specific CD4^+^ T cells ex vivo with a panel of 14 MHC class II tetramers that target T cells restricted by 8 common HLA‐DR molecules.^5^, ^29^ The ability to analyze T cells ex vivo is a significant advantage over other contemporary techniques that involve prolonged culture of T cells with allergen, and/or analysis of bulk CD4^+^ T‐cell population, which may not accurately reflect their state in vivo. However, analysis of rare cell populations also has inherent weaknesses related to the small number of events detected and thus the conclusions drawn from this analysis require replication in further studies.

In this study, treatment with peptide immunotherapy was not associated with any change in the peripheral blood frequency of total allergen‐specific (tetramer^+^) CD4^+^ T cells. Furthermore, specific analysis of tetramer^+^CD27^− ^CD4^+^ T cells that are thought to be important in disease pathogenesis and to be deleted during subcutaneous immunotherapy (SCIT) with allergen extracts did not reveal any significant changes in these subsets. Thus, our findings identify a potentially significant difference in mechanism between traditional immunotherapy approaches with intact allergens, and immunotherapy with short peptide sequences containing T‐cell epitopes. Subcutaneous treatment with alder pollen extract^23^ or with Timothy grass pollen extract^24^ has been reported to result in the disappearance of terminally differentiated effector memory CD4^+^ T cells (CRTh2^+^CD27^−^) from the peripheral blood. The reasons for this difference remain unclear, although SCIT is performed over a relatively long period of time with multiple injections and a high cumulative dose of allergen. Peptide immunotherapy on the other hand is given via a different route (intradermal vs subcutaneous) and with fewer (4‐8) doses. It is interesting to note that in murine models of peptide immunotherapy, higher doses of peptide have been associated with deletion of antigen‐specific CD4^+^ T cells,^33^ and thus, higher dose regimens of Cat‐PAD may result in deletion. A similar deletion effect may be responsible for the difference in efficacy of 3 nmol (used in this study) vs 6 nmol Cat‐PAD at 54 weeks and beyond.^2^ At the 24 weeks (the time point employed in this study), both the 3 and 6 nmol doses display identical reductions in TNSS; however, only the 6 nmol dose of Cat‐PAD retains its therapeutic effect after 54 weeks. It may be that the prolonged therapeutic effect of the 6nmol dose may be due to the deletion of Fel d 1‐specific CD4^+^ T cells. Another potential important difference is the nature of the allergen to which the subject is allergic. Although little is known regarding the differences in immune responses to cat dander vs Timothy grass vs alder pollen, these three allergens may result in different immunological changes.

A potential weakness of this and other studies of antigen‐specific CD4^+^ T‐cell frequencies is that, as a result of the low frequency of tetramer+ T cells in peripheral blood and the wide variability in the range of baseline frequencies observed, few if any studies are adequately statistically powered to detect subtle changes that may, nonetheless, be biologically important. Flow cytometric analysis of tetramer+ T cells in this and other studies is based on capture of only a small number of events. As a result, we can only conclude with confidence that substantial changes in frequency did not occur following peptide immunotherapy. A further weakness of the study is the use of peripheral blood which may not be representative of treatment‐induced changes in other compartments such as the lymphatics and the target organs.

Historically, whole allergen immunotherapy (eg, SCIT) has been associated with a shift in the response to allergen from Th2 to Th1, a phenomenon referred to as “immune deviation”.^34^, ^35^, ^36^ Previous studies of peptide immunotherapy in cat allergy provided some evidence for increased Th1 responses in the skin following allergen challenge^37^ but several related studies were unable to demonstrate evidence of immune deviation in peripheral blood responses to allergen.^38^, ^39^ Neither skin nor peripheral blood data from these studies focused on allergen‐specific CD4^+^ T cells per se. In the current study, we have used MHC class II tetramers to focus specifically on Fel d 1‐specific CD4^+^ T cells. We used chemokine receptors as surrogate markers of Th1 (CXCR3) and Th2 (CRTh2) subsets and compared the proportions of each before and after treatment with Fel d 1 synthetic peptides. However, these markers are only representative of Th1 and Th2 but do not define them. Thus, our analysis should be considered an estimate of changes in the Th1 and Th2 compartments. In agreement with earlier peptide immunotherapy studies, we did not see any evidence of a Th2 to Th1 shift in the allergen‐specific CD4^+^ T‐cell response in the peripheral blood. We conclude that immune deviation, at least at the level of peripheral blood responses to allergen, is unlikely to be a major mechanism of action of peptide immunotherapy.

There was a significant difference in the decrease in surface levels of CRTh2 on Fel d 1‐specific CD4^+^ T cells when comparing subjects treated with peptide immunotherapy vs placebo. CRTh2 is the receptor for prostaglandin D_2_ (PGD_2_), which is produced by mast cells upon crosslinking of IgE on their surface, and is expressed primarily by Th2 cells.^40^, ^41^ The ligation of CRTh2 by PGD_2_ serves as a chemoattractant signal for CD4^+^ T cells and also facilitates the production of cytokines by Th2 cells.^42^ Antagonism of PGD_2_ binding to CRTh2 has been achieved with small molecules that have been shown to be effective in reducing symptoms of allergic disease following allergen provocation.^42^, ^43^, ^44^ Thus, decreased surface expression of CRTh2 by allergen‐specific CD4^+^ T cells following peptide immunotherapy may serve to limit both the recruitment of Fel d 1‐specific CD4^+^ T cells to sites of allergen contact and their production of Th2 cytokines. Studies in mice have demonstrated that downregulation of chemokine receptors can inhibit leukocyte recruitment.^45^ Few studies have assessed the effect of SIT on CRTh2 expression by CD4^+^ T cells. One such study was conducted by Wambre et al in individuals that had completed at least 3 years of SIT for Alder pollen allergy. In that study, the authors found a reduction in the percentage of Alder pollen‐specific CD4^+^ T cells expressing CRTh2, along with other markers of Th2 cells.^23^ Although we did not find a reduction in the percentage of Fel d 1‐specific CD4^+^ T cells expressing CRTh2, the decrease in its level of expression is in line with the idea that SIT can affect the expression of CRTh2 by allergen‐specific CD4^+^ T cells. However, further exploration with adequately powered studies will be required to elucidate whether PIT significantly alters the expression of CRTh2 on Fel d 1‐specific CD4^+^ T cells and what the exact functional consequence this decrease might be.

In conclusion, peptide immunotherapy was not associated with substantial deletion of allergen‐specific CD4^+^ T cells, including the CD27 subpopulation that has recently been implicated in the pathogenesis of allergic disease. A specific reduction in surface levels of the PGD_2_ receptor CRTh2 was observed. We hypothesize that downregulation of CRTh2 might render allergen‐specific CD4^+^ T cells relatively unresponsive to PGD_2_ gradients emanating from mast cells activated at the site of allergen exposure. This could result in a failure to recruit and activate these cells, thereby reducing Th2 inflammatory responses in the airways.

## CONFLICTS OF INTEREST

ML is a co‐Founder of Circassia Ltd, the company that sponsored the clinical trial that generated the blood samples employed in this study. ML is a consultant to Circassia Ltd and an associated company Adiga Life Sciences Inc ML is a shareholder in Circassia Pharmaceuticals PLC and Adiga Life Sciences Ltd. ML is a consultant to Aravax Pty. All of these companies are involved in the development of peptide immunotherapy for the treatment of allergic diseases. The other authors declare no conflicts of interest relevant to the subject of this study.

## AUTHOR CONTRIBUTIONS

CDR contributed to study design, performed experimental work, analyzed data, developed data figures, and contributed to writing the manuscript; ET analyzed data, developed data figures, and contributed to writing the manuscript; EJ designed and generated tetramer reagents and contributed to writing the manuscript; WWK designed and generated tetramer reagents and contributed to writing the manuscript; ML designed the study, analyzed data, developed data figures, and contributed to writing the manuscript.
