# Supplementary figures and images for: Modulation of CRTh2 expression on allergen‐specific T cells following peptide immunotherapy
Source: Allergy. 2019 Jun 7;74(11):2157–66. doi: 10.1111/all.13867 (PMC6817377; doi:10.1111/all.13867)

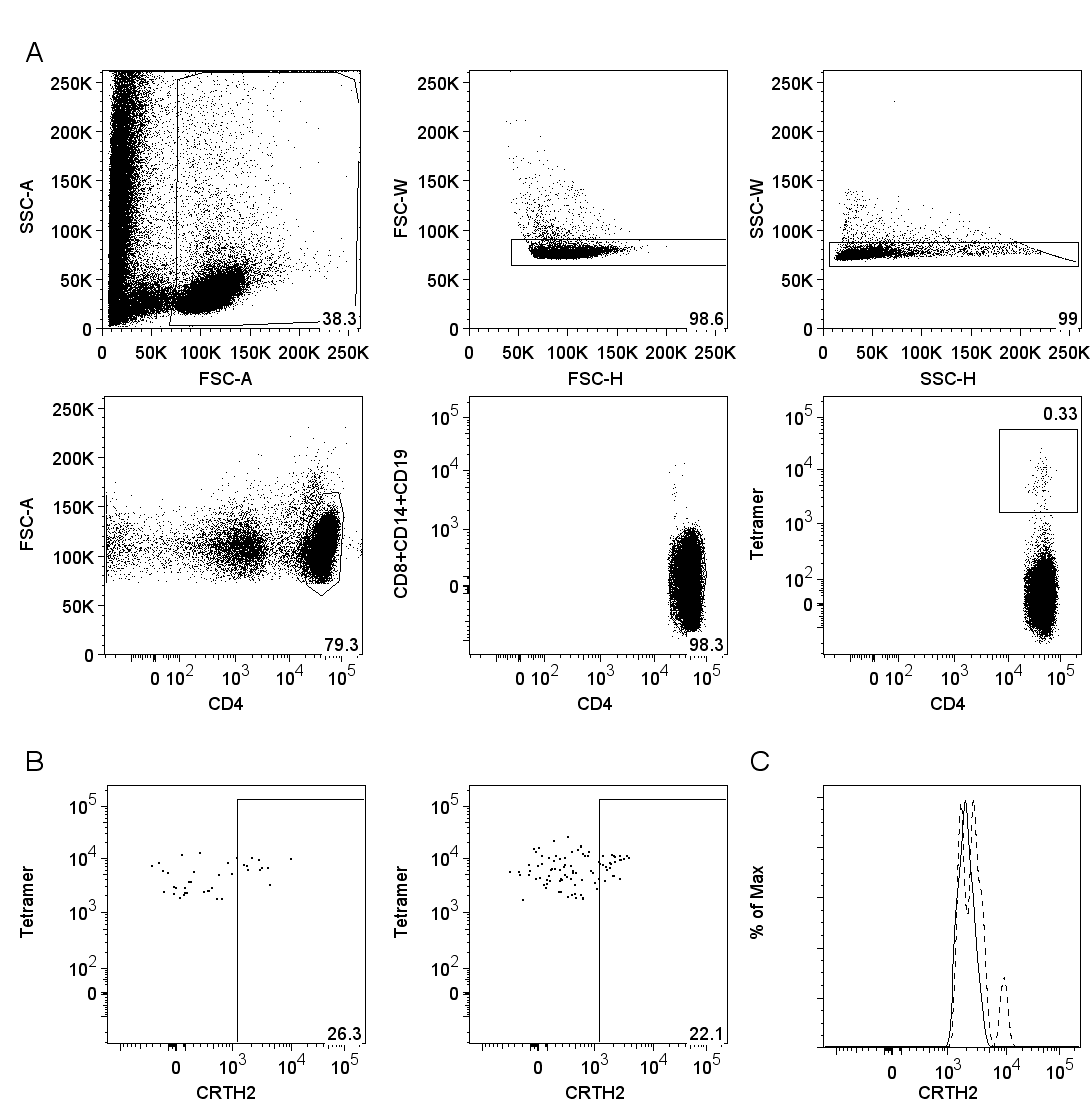

Supplement: Supplementary file 2 [file ALL-74-2157-s002.tif]
